# *XRCC5/6* polymorphisms and their interactions with smoking, alcohol consumption, and sleep satisfaction in breast cancer risk: A Chinese multi‐center study

**DOI:** 10.1002/cam4.3847

**Published:** 2021-03-18

**Authors:** Li‐Xiang Yu, Li‐Yuan Liu, Yu‐Juan Xiang, Fei Wang, Fei Zhou, Shu‐Ya Huang, Chao Zheng, Chun‐Miao Ye, Wen‐Zhong Zhou, Geng‐Shen Yin, Jia‐Lin Zhang, Shu‐De Cui, Fu‐Guo Tian, Zhi‐Min Fan, Cui‐Zhi Geng, Xu‐Chen Cao, Zhen‐Lin Yang, Xiang Wang, Hong Liang, Shu Wang, Hong‐Chuan Jiang, Xue‐Ning Duan, Hai‐Bo Wang, Guo‐Lou Li, Qi‐Tang Wang, Jian‐Guo Zhang, Feng Jin, Jin‐Hai Tang, Liang Li, Shi‐Guang Zhu, Wen‐Shu Zuo, Zhong‐Bing Ma, Zhi‐Gang Yu

**Affiliations:** ^1^ Department of Breast Surgery The Second Hospital Cheeloo College of Medicine Shandong University Jinan China; ^2^ Institute of Translational Medicine of Breast Disease Prevention and Treatment Shandong University Jinan China; ^3^ Department of Breast Surgery Affiliated Tumor Hospital of Zhengzhou University Zhengzhou China; ^4^ Department of Breast Surgery Shanxi Cancer Hospital Taiyuan China; ^5^ Department of Breast Surgery The First Hospital of Jilin University Changchun China; ^6^ Department of Breast Center The Fourth Hospital of Hebei Medical University Shijiazhuang China; ^7^ Department of Breast Surgery Tianjin Medical University Cancer Institute and Hospital Tianjin China; ^8^ Department of Thyroid and Breast Surgery The First Affiliated Hospital of Binzhou Medical University Binzhou China; ^9^ Department of Breast Surgery Cancer Hospital Chinese Academy of Medical Sciences Beijing China; ^10^ Department of General Surgery Linyi People's Hospital Linyi China; ^11^ Department of Breast Disease Center Peking University People's Hospital Beijing China; ^12^ Department of General Surgery Beijing Chaoyang Hospital Beijing China; ^13^ Department of Breast Disease Center Peking University First Hospital Beijing China; ^14^ Department of Breast Center Qingdao University Affiliated Hospital Qingdao China; ^15^ Department of Breast and Thyroid Surgery Weifang Traditional Chinese Hospital Weifang China; ^16^ Department of Breast Surgery Qingdao Central Hospital Qingdao China; ^17^ Department of General Surgery The Second Affiliated Hospital of Harbin Medical University Harbin China; ^18^ Department of Breast Surgery The First Affiliated Hospital of China Medical University Shenyang China; ^19^ Department of General Surgery Nanjing Medical University Affiliated Cancer Hospital Cancer Institute of Jiangsu Province Nanjing China; ^20^ Department of Breast and Thyroid Surgery Zibo Central Hospital Zibo China; ^21^ Department of Breast Surgery Yantai Yuhuangding Hospital Yantai China; ^22^ Department of Breast Cancer Center Shandong Cancer Hospital Jinan China

**Keywords:** alcohol consumption, breast cancer, gene‐environment interaction, sleep satisfaction, smoking, *XRCC5*, *XRCC6*

## Abstract

**Background:**

X‐ray repair cross‐complementary 5 (*XRCC5*) and 6 (*XRCC6*) are critical for DNA repair. Few studies have assessed their association with breast cancer risk, and related gene‐environment interactions remain poorly understood. This study aimed to determine the influence of *XRCC5*/*6* polymorphisms on breast cancer risk, and their interactions with cigarette smoking, alcohol consumption, and sleep satisfaction.

**Methods:**

The study included 1039 patients with breast cancer and 1040 controls. Four single‐nucleotide polymorphisms of *XRCC5* and two of *XRCC6* were genotyped. Information about smoking, alcohol consumption, and sleep satisfaction was collected through questionnaires. Odds ratios (OR) and related 95% confidence intervals (95% CI) were assessed using unconditional logistic regression models. Gene‐environment interactions were analyzed using logistic regression with multiplicative interaction models.

**Results:**

*XRCC5* rs16855458 was associated with increased breast cancer risk in the co‐dominant (*p*_trend_ = 0.003) and dominant (CA + AA vs. CC, OR = 1.29, 95% CI = 1.07–1.56, *p* = 0.008) genetic models after Bonferroni correction. The CG + GG genotype of *XRCC6* rs2267437 was associated with an increased risk of estrogen receptor‐negative/progesterone receptor‐negative (ER−/PR−) breast cancer (CG + GG vs. CC: OR = 1.54, 95% CI = 1.12–2.13, *p* = 0.008) after Bonferroni correction. Moreover, an antagonistic interaction between *XRCC5* rs16855458 and alcohol consumption (*p*_interaction_ = 0.017), and a synergistic interaction between *XRCC6* rs2267437 and sleep satisfaction were associated with breast cancer risk (*p*_interaction_ = 0.0497). However, these interactions became insignificant after Bonferroni correction.

**Conclusion:**

*XRCC5* rs16855458 was associated with breast cancer risk, and *XRCC6* rs2267437 was associated with the risk of ER−/PR− breast cancer. Breast cancer risk associated with *XRCC5* and *XRCC6* polymorphisms might vary according to alcohol consumption and sleep satisfaction, respectively, and merit further investigation.

## Graphical abstract

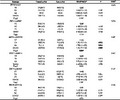

## 1 INTRODUCTION

The identification of risk factors and susceptible populations of breast cancer has become imperative in China given the recent marked increase of breast cancer cases among Chinese women.^1^, ^2^ Epidemiological studies identified cigarette smoking, alcohol consumption, and sleep disorders among the several environmental risk factors for breast cancer.^3^, ^4^, ^5^ Nevertheless, only a small proportion of exposed people develop breast cancer, suggesting that breast cancer incidence is not only correlated with environmental factors, but also with genetic susceptibility. Gene‐environment interactions can be deterministic for breast cancer development in individuals who were exposed to environmental risk factors. However, the underlying genetic mechanisms of breast carcinogenesis, especially gene‐environment interactions, remain poorly understood.

Cells under the influence of endogenous and exogenous factors in vivo sustain a large number of DNA‐damaging events daily.^6^ X‐ray repair cross‐complementing 5 (*XRCC5*) and 6 (*XRCC6*) are key genes of the non‐homologous end joining (NHEJ) repair pathway, which is an important DNA repair mechanism. *XRCC5*/*6* polymorphisms correlate with susceptibility to develop various malignant tumors.^7^, ^8^ *XRCC5* rs16855458 and rs9288516 have potential biological functions as they cause changes in the binding sites of transcription factors, and correlate with risk for hepatocellular carcinoma,^9^, ^10^ whereas *XRCC6* rs2267437 and *XRCC5* rs3835 were correlated with breast cancer risk in European women.^11^ However, relatively few studies have investigated *XRCC5*/*6* polymorphisms and breast cancer susceptibility in Chinese women, and available results have been inconsistent. For example, a study conducted in Taiwan found that *XRCC5* rs3835 was not linked to breast cancer susceptibility,^12^ contrasting with the findings of a previous European study.^11^ These inconsistencies might be due to the specific genetic landscape of different populations. Moreover, gene‐environment interactions in the occurrence of breast cancer have been largely ignored. Smoking, alcohol consumption, and sleep disorders are considered important environmental carcinogenic factors, and DNA damage caused by these factors is an important underlying mechanism.^13^, ^14^, ^15^ DNA repair pathways are critical to maintain DNA stability and prevent long‐lasting DNA damage caused by environmental exposure.^16^ Therefore, polymorphisms on DNA repair genes might interact with these environmental factors during carcinogenesis. Several studies reported interactions between polymorphisms of DNA repair genes such as *XRCC1* and smoking in the etiology of breast cancer.^17^ However, few studies investigated potential interactions between *XRCC5*/*6* polymorphisms and carcinogenesis‐related environmental factors, especially in Chinese mainland populations. Relevant investigations will contribute to breast cancer risk assessment and the development of intervention strategies.

The present study aimed to clarify the effects of *XRCC5*/*6* polymorphisms on breast cancer susceptibility among Chinese women and their potential interactions with smoking, alcohol consumption, and sleep satisfaction.

## 2 MATERIALS AND METHODS

### 2.1 Participants

The study participants have been previously described.^18^ Briefly, participants in the case group had newly diagnosed, histologically confirmed breast cancer and were recruited at 21 hospitals in Northern and Eastern China between April 2012 and April 2013. The control group comprised age‐matched (±3 years) volunteers recruited at the same hospital who were examined within 2 months of the case group and were confirmed as being breast cancer free by negative physical and imaging findings. Participants with other malignant tumors were excluded from the study. The ethics committee of the Second Hospital, Cheeloo College of Medicine, Shandong University had approved this study, and all participants signed informed consent.

### 2.2 Data collection

Demographic information and lifestyle habits of the participants were obtained using a structured questionnaire as described previously.^19^ Smokers were defined as those who continuously or cumulatively smoked cigarettes for at least 6 months. Drinkers were defined as those who consumed alcohol at least once a month for at least 6 months. Sleep satisfaction was determined as one of the four responses to the question, “In the most recent 1‐year period, have you been satisfied with your sleep?” The choices were, “very satisfied,” “satisfied,” “dissatisfied,” and “very dissatisfied.” The estrogen receptor (ER) and progesterone receptor (PR) status were determined using immunohistochemical staining and obtained from the medical records of the patients. According to the American Society of Clinical Oncology/College of American Pathologists (2010) guideline recommendations, ER and PR positivity was defined as ≥1% of tumor cells with positive staining.^20^

### 2.3 Genotyping

Fasting blood samples were collected into EDTA Vacutainers (Becton Dickinson and Co.) and stored at −80°C after sedimentation. DNA was extracted using the Wizard Genomic DNA Purification Kit (A1120, Promega). Single‐nucleotide polymorphisms (SNPs) were selected based on their reported association with cancer risk, and with a minor allelic frequency (MAF) >5% according to the 1000 Genomes project (https://www.ncbi.nlm.nih.gov/snp/). rs3835, rs828907, rs16855458, and rs9288516 of *XRCC5*,^9^, ^11^, ^21^, ^22^ and rs2267437 and rs5751131 of *XRCC6* ^9^, ^11^ were selected for further analysis (Table 1). All participants were genotyped using the Sequenom MassARRAY SNP system (CapitalBio Technology), as previously described.^18^ Double‐distilled water was used as a negative control. Tests were repeated on 5% of randomly selected samples as quality control, and 100% consistency was achieved.

**TABLE 1:** Distribution of target SNPs of *XRCC5*/*6*

Gene	SNP IDs	Chromosome	Location in gene region	Base change	MAF
*XRCC5*	rs3835	2	Intron variant	G > A	0.206
*XRCC5*	rs828907	2	2 KB upstream variant	G > T	0.326
*XRCC5*	rs16855458	2	Intron variant	C > A	0.149
*XRCC5*	rs9288516	2	Intron variant	T > A	0.152
*XRCC6*	rs2267437	22	2 KB upstream variant	C > G	0.260
*XRCC6*	rs5751131	22	Intron variant	A > G	0.107

Abbreviations: MAF, minor allele frequency; SNP, single‐nucleotide polymorphism.

### 2.4 Statistical analysis

Differences in demographic and lifestyle data between the case and control groups were assessed by *χ* ^2^ tests. A population representative was detected using Hardy–Weinberg equilibrium (HWE) in the control group. Associations between co‐dominant (wild‐type homozygous vs. heterozygous vs. mutant homozygous) and dominant (wild‐type homozygous vs. heterozygous and mutant homozygous) models of genetic variants and breast cancer were assessed using unconditional logistic regression. Odds ratios (OR) with 95% confidence interval (95% CI) were estimated after adjustment for age, body mass index (BMI), menstrual status, and family history of breast cancer. Trends were tested by considering the categorical genotypes as continuous variables in co‐dominant inheritance profiles. Gene‐environment interactions were assessed using logistic regression including multiplicative interaction models. Sleep satisfaction was defined as “dissatisfied” versus “satisfied,” and the dominant model with the homozygous common genotypes was adopted as the reference to facilitate the interaction analysis. The Bonferroni correction was employed for multiple testing and the significant *p* value was set at 0.0083 (0.05/6) for testing the six loci. All data were statistically analyzed using SPSS 23.0 (IBM Corp.).

## 3 RESULTS

### 3.1 Demographic and lifestyle characteristics of the participants

Table 2 lists the demographic and lifestyle characteristics of the 1039 cases and 1040 controls. Menstrual status, BMI, family history of breast cancer, smoking, and sleep satisfaction considerably differed between the groups, whereas age and alcohol consumption did not.

**TABLE 2:** Demographic and lifestyle characteristics of the participants

Variable^a^	Control, *n* (%)	Case, *n* (%)	*χ* ^2^	*p* ^b^
Age, y				
25–44	440 (42.3)	392 (37.7)	5.75	0.057
45–59	523 (50.3)	550 (52.9)		
60–70	77 (7.4)	97 (9.3)		
BMI			10.00	0.007
<24	515 (51.0)	481 (48.1)		
24–27.9	407 (40.3)	388 (38.8)		
≥28	88 (8.7)	131 (13.1)		
Menstrual status			9.00	0.003
Premenopausal	724 (72.5)	667 (66.4)		
Postmenopausal	274 (27.5)	338 (33.6)		
Family history of breast cancer			4.13	0.042
No	995 (97.4)	983 (95.7)		
Yes	27 (2.6)	44 (4.3)		
Smoking				
No	1016 (98.1)	1000 (96.6)	4.22	0.040
Yes	20 (1.9)	35 (3.4)		
Alcohol consumption			2.50	0.114
No	935 (90.3)	910 (88.1)		
Yes	101 (9.7)	123 (11.9)		
Sleep satisfaction			8.08	0.004
Satisfied	874 (85.9)	829 (81.3)		
Dissatisfied	143 (14.1)	191 (18.7)		

Abbreviations: BMI, body mass index.

^a^ The data were presented in the form of classified variables.

^b^ *p* value was calculated by the *χ* ^2^ test, and the *p* < 0.05 was statistically significant.

### 3.2 Genotype distribution of *XRCC5*/6 and breast cancer risk

Table 3 shows the effects of *XRCC5*/*6* SNP genotypes and their adjusted effects on breast cancer risk. All SNPs followed the Hardy–Weinberg equilibrium (*p* > 0.05). Logistic regression analyses showed that *XRCC5* rs16855458 was associated with increased breast cancer risk in the co‐dominant genetic model (*p*_trend_ = 0.003), and the dominant genetic model (CA + AA vs. CC: OR = 1.29, 95% CI = 1.07–1.56, *p* = 0.008) after the Bonferroni correction. The other five SNPs were not significantly associated with breast cancer risk.

**TABLE 3:** Genotype distribution of *XRCC5*/6 and breast cancer risk

Genotype	Control, *n* (%)	Case, *n* (%)	OR (95%CI)^a^	*p*	HWE^b^
*XRCC5* rs3835					0.173
GG	855 (84.7)	866 (85.6)	1(ref)		
GA	151 (15.0)	140 (13.8)	0.93 (0.72–1.21)	0.587	
AA	3 (0.3)	6 (0.6)	3.06 (0.61–15.27)	0.173	
GA + AA	154 (15.3)	146 (14.4)	0.96 (0.74–1.24)	0.756	
*p*_trend_ ^c^			0.965		
*XRCC5* rs828907					0.056
GG	636 (62.9)	636 (62.9)	1(ref)		
GT	344 (34.0)	328 (32.4)	0.92 (0.76–1.12)	0.416	
TT	31 (3.1)	47 (4.6)	1.41 (0.87–2.29)	0.159	
GT + TT	375 (37.1)	375 (37.1)	0.96 (0.80–1.17)	0.708	
*p*_trend_ ^c^			0.833		
*XRCC5* rs16855458					0.287
CC	652 (64.4)	585 (57.8)	1(ref)		
CA	328 (32.4)	372 (36.8)	1.24 (1.02–1.51)	**0.031**	
AA	33 (3.3)	55 (5.4)	1.78 (1.12–2.82)	**0.014**	
CA + AA	361 (35.6)	427 (42.2)	1.29 (1.07–1.56)	**0.008**	
*p*_trend_ ^c^			**0.003**		
*XRCC5* rs9288516					0.488
TT	276 (27.2)	314 (30.9)	1 (ref)		
TA	516 (50.9)	485 (47.8)	0.82 (0.66–1.02)	0.072	
AA	221 (21.8)	216 (21.3)	0.87 (0.67–1.12)	0.280	
TA + AA	737 (72.8)	701 (69.1)	0.84 (0.68–1.02)	0.080	
*p*_trend_ ^c^			0.222		
*XRCC6* rs2267437					0.247
CC	628 (61.9)	623 (61.8)	1 (ref)		
CG	332 (32.7)	329 (32.6)	1.03 (0.84–1.25)	0.803	
GG	54 (5.3)	56 (5.6)	0.95 (0.64–1.43)	0.818	
CG + GG	386 (38.1)	385 (38.2)	1.02 (0.84–1.22)	0.879	
*p*_trend_ ^c^			0.987		
*XRCC6* rs5751131					0.251
AA	341 (33.9)	328 (33.1)	1 (ref)		
AG	474 (47.1)	490 (49.4)	1.17 (0.95–1.44)	0.134	
GG	191 (19.0)	173 (17.5)	0.97 (0.74–1.26)	0.797	
AG + GG	665 (66.1)	663 (66.9)	1.11 (0.92–1.35)	0.288	
*p*_trend_ ^c^			0.878		

Bold values indicate *p* < 0.05.

Abbreviations: CI, Confidence Interval; HWE, Hardy‐Weinberg equilibrium; OR, Odds Ratio; ref, reference.

^a^ Adjusted for age, BMI, menstrual status, and family history of breast cancer.

^b^ *p* value for Hardy‐Weinberg equilibrium.

^c^ *p* trend of the co–dominant inheritance patterns.

### 3.3 Associations between *XRCC5*/*6* polymorphisms and risk of ER+/PR+, ER−/PR− breast cancer

Among the 1039 cases, 918 (88.4%) cases had explicit joint ER and PR statuses. Overall, 614 (59.1%) cases were ER+/PR+, 212 (20.4%) were ER−/PR−, 77 (7.4%) were ER+/PR−, and 15 (1.4%) cases were ER−/PR+. Due to the limited number of ER+/PR− and ER−/PR+ cases, they were excluded from further analysis. Table 4 shows the associations between the SNPs genotypes of *XRCC5*/*6* and the risk of ER+/PR+, ER−/PR− breast cancer. The CG + GG genotype of *XRCC6* rs2267437 was associated with an increased risk of ER−/PR− breast cancer (CG + GG vs. CC: OR = 1.54, 95% CI = 1.12–2.13, *p* = 0.008) after Bonferroni correction. Increased ER+/PR+ breast cancer risk in the co‐dominant genetic model of *XRCC5* rs16855458 after Bonferroni correction was observed (*p*_trend_ = 0.008). The AA genotype of *XRCC5* rs16855458 was associated with an increased risk of both ER+/PR+ and ER−/PR− breast cancer (AA vs. CC: for ER+/PR+, OR = 1.84, 95% CI = 1.10–3.07, *p* = 0.019; for ER−/PR−, OR = 2.25, 95% CI = 1.14–4.46, *p* = 0.019), but not after Bonferroni correction. The other SNPs were not significantly associated with ER+/PR+ or ER−/PR− breast cancer risk.

**TABLE 4:** Associations between *XRCC5*/*6* polymorphisms and risk of ER+/PR+, ER−/PR− breast cancer

Genotype	Control, *n* (%)	ER+/PR+			ER−/PR−		
		Case, *n* (%)	OR (95%CI)^a^	*p*	Case, *n* (%)	OR (95%CI)^a^	*p*
*XRCC5* rs3835							
GG	855 (84.7)	520 (86.5)	1 (ref)		171 (83.0)	1 (ref)	
GA	151 (15.0)	77 (12.8)	0.89 (0.65–1.20)	0.434	34 (16.5)	1.17 (0.77–1.80)	0.463
AA	3 (0.3)	4 (0.7)	3.46 (0.62–19.2)	0.156	1 (0.5)	2.98 (0.27–33.42)	0.375
GA + AA	154 (15.3)	81 (13.5)	0.92 (0.68–1.24)	0.589	35 (17.0)	1.20 (0.79–1.82)	0.404
*p*_trend_ ^b^			0.796			0.350	
*XRCC5* rs828907							
GG	636 (62.9)	375 (62.3)	1 (ref)		129 (62.3)	1 (ref)	
GT	344 (34.0)	196 (32.6)	0.92 (0.73–1.15)	0.457	74 (35.7)	1.02 (0.73–1.42)	0.903
TT	31 (3.1)	31 (5.1)	1.52 (0.89–2.60)	0.124	4 (1.9)	0.49 (0.15–1.65)	0.250
GT + TT	375 (37.1)	227 (37.7)	0.97 (0.78–1.21)	0.783	78 (37.7)	0.98 (0.70–1.35)	0.880
*p*_trend_ ^b^			0.733			0.616	
*XRCC5* rs16855458							
CC	652 (64.4)	351 (58.1)	1 (ref)		125 (61.0)	1 (ref)	
CA	328 (32.4)	217 (35.9)	1.23 (0.98–1.45)	0.077	67 (32.7)	1.08 (0.77–1.52)	0.660
AA	33 (3.3)	36 (6.0)	1.84 (1.10–3.07)	**0.019**	13 (6.3)	2.25 (1.14–4.46)	**0.019**
CA + AA	361 (35.6)	253 (41.9)	1.29 (1.04–1.60)	**0.023**	89 (39.0)	1.19 (0.86–1.65)	0.294
*p*_trend_ ^b^			**0.008**			0.089	
*XRCC5* rs9288516							
TT	276 (27.2)	192 (31.7)	1 (ref)		63 (30.6)	1 (ref)	
TA	516 (50.9)	284 (46.9)	0.79 (0.62–1.02)	0.066	101 (49.0)	0.81 (0.56–1.16)	0.243
AA	221 (21.8)	129 (21.3)	0.85 (0.63–1.15)	0.294	42 (20.4)	0.79 (0.51–1.24)	0.310
TA + AA	737 (72.8)	413 (68.3)	0.81 (0.64–1.02)	0.076	143 (69.4)	0.80 (0.57–1.13)	0.204
*p*_trend_ ^b^			0.230			0.273	
*XRCC6* rs2267437							
CC	628 (61.9)	384 (63.8)	1 (ref)		107 (52.2)	1 (ref)	
CG	332 (32.7)	189 (31.4)	0.95 (0.75–1.20)	0.657	87 (42.4)	1.66 (1.20–2.31)	**0.002**
GG	54 (5.3)	29 (4.8)	0.87 (0.54–1.39)	0.551	11 (5.4)	0.89 (0.41–1.93)	0.762
CG + GG	386 (38.1)	218 (36.2)	0.94 (0.75–1.17)	0.556	98 (47.8)	1.54 (1.12–2.13)	**0.008**
*p*_trend_ ^b^			0.496			0.063	
*XRCC6* rs5751131							
AA	341 (33.9)	185 (31.6)	1 (ref)		73 (36.0)	1 (ref)	
AG	474 (47.1)	303 (51.7)	1.24 (0.97–1.57)	0.083	94 (46.3)	1.07 (0.75–1.53)	0.693
GG	191 (19.0)	98 (16.7)	0.96 (0.70–1.31)	0.783	36 (17.7)	0.90 (0.56–1.43)	0.645
AG + GG	665 (66.1)	401 (68.4)	1.16 (0.92–1.45)	0.214	130 (64.0)	1.02 (0.73–1.43)	0.900
*p*_trend_ ^b^			0.839			0.765	

Bold values indicate *p* < 0.05.

Abbreviations: CI, Confidence Interval; ER, estrogen receptor; OR, Odds Ratio; PR, progesterone receptor; ref, reference.

^a^ Adjusted for age, BMI, menstrual status, and family history of breast cancer.

^b^ *p* trend of the co‐dominant inheritance patterns.

### 3.4 Interactions between *XRCC5*/*6* polymorphisms and smoking, alcohol consumption, and sleep satisfaction in breast cancer risk

The effects of potential interactions between target (*XRCC5* and *XRCC6)* SNPs and smoking, alcohol consumption, and sleep satisfaction on breast cancer risk were analyzed. An antagonistic interaction was found between *XRCC5* rs16855458 and alcohol consumption (*p*_interaction_ = 0.017). Compared with nondrinkers carrying the rs16855458 CC genotype, risk for breast cancer was increased in nondrinkers harboring the CA + AA genotype (OR = 1.41, 95% CI = 1.16–1.72, *p* = 0.001) and drinkers with the CC genotype (OR = 1.63, 95% CI = 1.12–2.38, *p* = 0.011), but not in drinkers with CA + AA genotype (OR = 1.08, 95% CI = 0.66–1.75, *p* = 0.769). The *XRCC6* rs2267437 genotype synergistically interacted with sleep satisfaction (*p*_interaction_ = 0.0497). Compared with satisfied sleepers carrying the rs2267437 CC genotype, breast cancer risk increased in dissatisfied sleepers harboring the CG + GG genotype (OR = 1.72, 95% CI = 1.13–2.61, *p* = 0.011). However, these interactions became insignificant after the Bonferroni correction. The other SNPs evaluated did not show significant interactions with smoking, alcohol consumption, or sleep satisfaction (Table 5).

**TABLE 5:** Interactions between *XRCC5*/*6* polymorphisms and smoking, alcohol consumption, and sleep satisfaction in breast cancer risk

Genotype	Exposure^a^	Smoking				Alcohol consumption				Sleep satisfaction			
		Control, *n* (%)	Case, *n* (%)	OR (95%CI) ^b^	*p*	Control, *n* (%)	Case, *n* (%)	OR (95%CI) ^b^	*p*	Control, *n* (%)	Case, *n* (%)	OR (95%CI) ^b^	*p*
*XRCC5* rs3835													
GG	(−)	834 (83.0)	833 (82.6)	1 (ref)		764 (76.0)	764 (75.9)	1 (ref)		717 (72.5)	694 (69.9)	1 (ref)	
GA + AA	(−)	151 (15.0)	141 (14.0)	0.96 (0.74–1.24)	0.743	145 (14.4)	124 (12.3)	0.89 (0.68–1.17)	0.408	132 (13.3)	113 (11.4)	0.92 (0.69–1.22)	0.550
GG	(+)	17 (1.7)	29 (2.9)	1.61 (0.87–2.97)	0.131	87 (8.7)	96 (9.5)	1.12 (0.81–1.56)	0.482	119 (12.0)	154 (15.5)	1.30 (0.98–1.71)	0.068
GA + AA	(+)	3 (0.3)	5 (0.5)	1.64 (0.39–6.94)	0.503	9 (0.9)	22 (2.2)	2.20 (0.98–4.91)	0.055	21 (2.1)	32 (3.2)	1.50 (0.83–2.71)	0.184
*p*_interaction_ ^c^				0.937				0.086				0.517	
*XRCC5* rs828907													
GG	(−)	625 (62.1)	610 (60.6)	1 (ref)		580 (57.6)	563 (56.0)	1 (ref)		533 (53.9)	521 (52.5)	1 (ref)	
GT + TT	(−)	365 (36.2)	362 (35.9)	0.99 (0.81–1.20)	0.894	326 (32.4)	324 (32.2)	1.01 (0.83–1.24)	0.917	317 (32.1)	283 (28.5)	0.90 (0.73–1.11)	0.338
GG	(+)	8 (0.8)	23 (2.3)	2.85 (1.26–6.45)	0.012	52 (5.2)	70 (7.0)	1.47 (0.99–2.18)	0.053	86 (8.7)	104 (10.5)	1.22 (0.88–1.70)	0.230
GT + TT	(+)	9 (0.9)	12 (1.2)	1.18 (0.48–2.89)	0.711	49 (4.9)	49 (4.9)	0.91 (0.58–1.43)	0.690	52 (5.3)	84 (8.5)	1.46 (0.99–2.15)	0.057
*p*_interaction_ ^c^				0.161				0.114				0.297	
*XRCC5* rs16855458													
CC	(−)	640 (63.4)	566 (56.2)	1 (ref)		593 (58.8)	501 (49.8)	1 (ref)		541 (54.6)	462 (46.5)	1 (ref)	
CA + AA	(−)	351 (34.8)	407 (40.4)	1.29 (1.06–1.56)	**0.009**	317 (31.4)	387 (38.5)	1.41 (1.16–1.72)	**0.001**	308 (31.1)	343 (34.5)	1.27 (1.03–1.56)	**0.025**
CC	(+)	11 (1.1)	18 (1.8)	1.69 (0.78–3.66)	0.180	57 (5.6)	81 (8.1)	1.63 (1.12–2.38)	**0.011**	96 (9.7)	111 (11.2)	1.26 (0.92–1.73)	0.157
CA + AA	(+)	7 (0.7)	17 (1.7)	2.65 (1.09–6.46)	**0.032**	42 (4.2)	37 (3.7)	1.08 (0.66–1.75)	0.769	45 (4.5)	77 (7.8)	1.91 (1.26–2.89)	**0.002**
*p*_interaction_ ^c^				0.747				**0.017**				0.507	
*XRCC5* rs9288516													
TT	(−)	268 (26.6)	299 (29.6)	1 (ref)		243 (24.1)	273 (27.1)	1 (ref)		236 (23.8)	249 (25.0)	1 (ref)	
TA + AA	(−)	723 (71.7)	677 (67.0)	0.83 (0.68–1.02)	0.080	665 (65.9)	617 (61.1)	0.80 (0.65–0.99)	**0.042**	613 (61.9)	559 (56.1)	0.84 (0.67–1.04)	0.115
TT	(+)	6 (0.6)	13 (1.3)	1.86 (0.69–5.00)	0.217	33 (3.3)	40 (4.0)	0.92 (0.54–1.57)	0.762	36 (3.6)	60 (6.0)	1.37 (0.84–2.23)	0.204
TA + AA	(+)	12 (1.2)	22 (2.2)	1.52 (0.73–3.18)	0.258	68 (6.7)	79 (7.8)	1.07 (0.54–1.57)	0.729	105 (10.6)	129 (12.9)	1.10 (0.79–1.53)	0.566
*p*_interaction_				0.981				0.254				0.885	
*XRCC6* rs2267437													
CC	(−)	613 (60.7)	599 (59.7)	1 (ref)		570 (56.4)	541 (54.0)	1 (ref)		513 (51.8)	499 (50.5)	1 (ref)	
CG + GG	(−)	379 (37.5)	371 (37.0)	1.01 (0.83–1.22)	0.900	341 (33.8)	343 (34.2)	1.06 (0.86–1.29)	0.549	337 (34.0)	302 (30.5)	0.91 (0.74–1.12)	0.392
CC	(+)	13 (1.3)	21 (2.1)	1.62 (0.80–3.28)	0.179	56 (5.5)	77 (7.7)	1.40 (0.95–2.07)	0.080	97 (9.8)	116 (11.7)	1.11 (0.81–1.52)	0.537
CG + GG	(+)	5 (0.5)	13 (1.3)	2.41 (0.84–6.91)	0.111	43 (4.3)	41 (4.1)	1.05 (0.66–1.66)	0.844	44 (4.4)	72 (7.3)	1.72 (1.13–2.61)	**0.011**
*p*_interaction_ ^c^				0.576				0.244				**0.0497**	
*XRCC6* rs5751131													
AA	(−)	330 (32.9)	316 (32.0)	1 (ref)		306 (30.5)	288 (29.2)	1 (ref)		287 (29.1)	261 (26.9)	1 (ref)	
AG + GG	(−)	652 (65.1)	639 (64.7)	1.10 (0.90–1.34)	0.361	599 (59.8)	581 (58.9)	1.11 (0.90–1.36)	0.326	559 (56.7)	528 (54.3)	1.11 (0.90–1.38)	0.333
AA	(+)	11 (1.1)	12 (1.2)	1.11 (0.48–2.56)	0.814	34 (3.4)	39 (4.0)	1.25 (0.75–2.08)	0.384	48 (4.9)	62 (6.4)	1.38 (0.89–2.13)	0.153
AG + GG	(+)	9 (0.9)	21 (2.1)	2.44 (1.09–5.46)	**0.030**	63 (6.3)	78 (7.9)	1.40 (0.94–2.07)	0.097	92 (9.3)	121 (12.4)	1.47 (1.05–2.07)	**0.024**
*p*_interaction_ ^c^				0.237				0.990				0.894	

Bold values indicate *p* < 0.05.

Abbreviations: CI, confidence interval; OR, odds ratio.

^a^ Exposure for smoking: (−), no; (+), yes. Exposure for drinking: (−), no; (+), yes. Exposure for sleep satisfaction: (−), satisfied; (+), dissatisfied.

^b^ Adjusted for age, BMI, menstrual status, and family history of breast cancer.

^c^ *p* interaction was calculated by the test for multiplicative interaction.

## 4 DISCUSSION

This study aimed to determine the influence of *XRCC5* and *XRCC6* polymorphisms on breast cancer risk, and potential interactions with cigarette smoking, alcohol consumption, and sleep satisfaction. The data revealed that *XRCC5* rs16855458 was associated with increased breast cancer risk in the co‐dominant genetic model, and the CG + GG genotype of *XRCC6* rs2267437 was associated with an increased risk of ER−/PR− breast cancer, even after Bonferroni correction. Antagonistic interaction between *XRCC5* rs16855458 and alcohol consumption, and synergistic interaction between *XRCC6* rs2267437 and sleep satisfaction were also found to affect breast cancer risk. However, these interactions became insignificant after applying the Bonferroni correction.

NHEJ repair is a major mechanism responsible for mending mammalian DNA double‐strand breaks.^23^ The Ku70 and Ku80 proteins, respectively, encoded by *XRCC6* and *XRCC5*, form the Ku heterodimer that plays key roles in the NHEJ pathway; thereby regulating the DNA repair function.^24^ Of note, abnormal Ku70/80 levels correlate with the development of various malignant tumors.^25^, ^26^ However, relatively few studies have investigated relationships between *XRCC5* polymorphisms and tumor susceptibility. The present study showed that *XRCC5* rs16855458 polymorphism correlates with breast cancer risk. The function of the rs16855458 polymorphism remains unclear; however, the FASTSNP software revealed that it has the potential to change binding sites of transcription factors.^9^, ^10^ Indeed, Li et al.^9^ correlated the rs16855458 polymorphism with hepatocellular carcinoma risk, with individuals carrying the CA + AA genotype having lower risk than those with the CC genotype. In contrast, this study showed that individuals with the rs16855458 CA + AA genotype had a higher risk of breast cancer. Moreover, the rs16855458 CA + AA genotype identified by Li et al.^9^ was protective only among males, whereas in the present study all participants were female. These inconsistencies suggest that the relationship between rs16855458 polymorphism and cancer risk susceptibility differs among various tumors, and/or might be associated with sex differences. Of note, the protective effect of this polymorphism on hepatocellular carcinoma was limited to patients with hepatitis B virus infection,^9^ which relies on hepatitis B virus insertion during NHEJ for inducing hepatocellular carcinoma, differing from the pathogenesis mechanisms of breast cancer.^27^

Environmental factors are important triggers of DNA damage. Therefore, polymorphisms in genes that repair DNA damage may interact with environmental factors for promoting breast cancer development. Herein, an antagonistic interaction was identified between the *XRCC5* rs16855458 CA + AA genotype and alcohol consumption in breast cancer risk. This particular genotype was associated with a reduced risk for hepatocellular carcinoma.^9^ Alcohol metabolites in the liver, such as acetaldehyde and reactive oxygen species, are mutagenic and carcinogenic,^28^, ^29^ and are correlated with an increased risk of breast cancer. Hence, it is possible that the rs16855458 CA + AA genotype reduces the impact of alcohol on breast cancer risk through liver protection. However, the identified rs16855458/alcohol consumption interaction became insignificant after applying the Bonferroni correction: Thus, the role and effect of this specific *XRCC5* polymorphism warrant further investigation.

The *XRCC6* rs2267437 is located upstream of the CACCC box in the Ku70 promoter region.^30^ Changes in this sequence can interfere with transcription factor binding, thus affecting the Ku70 levels and the overall NHEJ repair process.^31^, ^32^ Several studies found an association between the rs2267437 polymorphism and breast cancer risk. Willems et al.^11^ reported a higher risk for breast cancer among women carrying the rs2267437 CG than those with the CC genotype. He et al.^33^ also reported similar findings with the rs2267437 CG or GG alleles in a Chinese female population. Herein, no statistically significant association between rs2267437 and the total risk of breast cancer was found. This inconsistency might be partly due to different genetic characteristics of the studied populations. To date, most studies exploring associations between rs2267437 polymorphisms and breast cancer risk included European populations,^11^, ^34^ and Chinese women have been evaluated in studies conducted mainly in Taiwan^12^ and Central China,^33^ covering relatively limited geographical areas and small samples. The present research was a multi‐center, large sample study encompassing East and North China, which offered a relatively better representation of the Chinese population. Another possible explanation for the observed data discrepancy could derive from complex gene‐environment interactions. Herein, a slightly synergistic interaction between the rs2267437 polymorphism and sleep satisfaction was reported to affect the occurrence of breast cancer before Bonferroni correction, supporting the importance of gene‐environment interaction. Sleep satisfaction is an important and necessary indicator of sleep quality and is considered to have an even more important effect than objective indicators on predicting sleep disorders and health.^35^, ^36^, ^37^ Night shift work and light exposure are important factors influencing sleep satisfaction,^37^ with working night shifts been reported to cause more DNA damage events,^14^ while low light exposure can reduce the DNA damage repair function by affecting melatonin release.^38^ Thus, sleep satisfaction could be associated with DNA damage events. Since *XRCC6* rs2267437 was associated with dysfunctional DNA damage repair,^31^, ^32^ a potential synergistic effect between rs2267437 and poor sleep patterns can further increase the risk of breast cancer. Although the interaction effect was not significant after Bonferroni correction, it also suggested a strong possible interaction effect as Bonferroni correction was a conservative and stringent correction test. Studies in larger patient cohorts would contribute to elucidating the clinical relevance of this interaction.

Several studies showed that DNA repair defects are associated with hormone receptor‐negative breast cancers. For example, the breast cancer susceptibility gene 1 (*BRCA1*), a tumor suppressor involved in homologous recombination pathways of DNA repair, was associated with the risk of hormone receptor‐negative breast cancer.^39^, ^40^ Moreover, polymorphisms of *XRCC4*, another essential gene in the NHEJ pathway, were found to be associated with PR− breast cancer risk.^41^ The present study revealed increased ER+/PR+ breast cancer risk in the co‐dominant genetic model of *XRCC5* rs16855458. However, the AA genotype of *XRCC5* rs16855458 was associated with an increased risk of both ER+/PR+ and ER−/PR− breast cancer. Therefore, the relationship between *XRCC5* rs16855458 and breast cancer risk by different hormone receptor states needs to be further explored. Furthermore, the CG + GG genotype of *XRCC6* rs2267437 was found to be associated with an increased risk of ER−/PR− breast cancer even after Bonferroni correction, but not with ER+/PR+ breast cancer. This finding demonstrates that this relationship might be genuine rather than a false positive. Polymorphisms of *XRCC6* rs2267437 were related to Ku70 protein expression, which was an essential protein in the NHEJ pathway of DNA double‐strand break.^30^, ^31^, ^32^ Studies had shown that the defects of DNA double‐strand break repair were associated with ER and PR negative breast cancer.^39^, ^41^ However, the specific mechanism between *XRCC6* rs2267437 variation and ER+/PR+ breast cancer risk is still unclear and further investigation is required.

This study has several limitations. The hospital‐based, case‐control design might hold an inherent selection bias. Also, only a few SNPs were selected for analysis, in particular only four variants of *XRCC5* and two of *XRCC6*, based on previous reports. Hence, some important *XRCC5*/*6* SNPs might have been missed. The sample size was also limited; thus, whether the results can be extrapolated to a wider area needs to be explored. Due to the lack of complete human epidermal growth factor receptor 2 (HER2) status data, this study only analyzed the correlation between *XRCC5*/*6* polymorphisms and the risk of breast cancer by different hormone receptor states, but not molecular subtypes. Nonetheless, the findings of this study are a good complement to the existing knowledge. In the future, better‐designed studies with larger samples, systematically selected SNPs, and molecular subtypes’ information are needed to further clarify the effects of gene‐environment interactions on the occurrence of breast cancer.

## 5 CONCLUSIONS

This study provides new evidence that *XRCC5* rs16855458 is associated with breast cancer risk among Chinese women, and that *XRCC6* rs2267437 is associated with the risk of ER−/PR− breast cancer. Moreover, potential interactions between *XRCC5* rs16855458 and alcohol consumption, and between *XRCC6* rs2267437 and sleep satisfaction were identified in relation to breast cancer risk. Despite that interactions became insignificant after conservative multiple‐comparison correction (Bonferroni correction), these results provide novel evidence for risk assessment and individual intervention for breast cancer in Chinese women.

## CONFLICT OF INTEREST

All authors declare no conflict of interest.

## Data Availability

The data that support the findings of this study are available from the corresponding author upon reasonable request.
